# Long non-coding RNA FAM230B is a novel prognostic and diagnostic biomarker for lung adenocarcinoma

**DOI:** 10.1080/21655979.2022.2034568

**Published:** 2022-03-15

**Authors:** Yu Cao, Hong Zhang, Jianming Tang, Rui Wang

**Affiliations:** Department of Thoracic Surgery, The Third Affiliated Hospital of Chongqing Medical University, Chongqing City, PR. China

**Keywords:** FAM230B, lung adenocarcinoma, diagnosis, prognosis

## Abstract

Long non-coding RNA (lncRNA) FAM230B has been reported to participate in gastric cancer and papillary thyroid cancer, while its role in other cancers has not been reported. We then explored the role of FAM230B in lung adenocarcinoma (LA). This study enrolled a total of 60 LA patients, 60 patients with gastric reflux disease (GRD), 60 cases of chronic obstructive pulmonary disease (COPD), 60 cases of asthma and 60 cases of healthy controls. LA and paired non-tumor tissues were donated by all LA patients. Plasma samples were donated by all participants. Expression of FAM230B in these samples was determined by RT-qPCR. The 60 LA patients were followed up for 5 years to evaluate the prognostic value of plasma FAM230B for LA. Diagnostic value of FAM230B for LA was analyzed with ROC curve analysis. FAM230B was highly expressed in LA tissues compared to that in non-tumor samples. In addition, plasma FAM230B was specifically upregulated in LA patients, but not in GRD, COPD and asthma patients. High expression levels of FAM230B in plasma samples were closely correlated with poor survival. Plasma FAM230B effectively separated LA patients from GRD, COPD, asthma and control groups. Plasma FAM230B was closely correlated with tumor size, but not other clinical factors of LA patients. Therefore, FAM230B is highly upregulated in LA and may serve as a potential diagnostic and prognostic biomarker for LA.

## Graphical abstract

**Figure:**
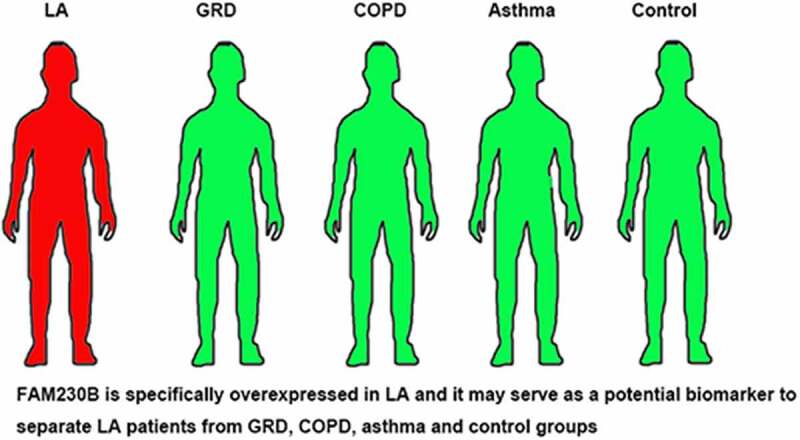

## Introduction

Lung adenocarcinoma (LA) is a type of aggressive tumor originates from bronchial mucosal glands that affects about 60 out of 100,000 people each year worldwide, with a higher incidence in men than that in women [1,2]. LA is classified as a common subtype of non-small cell lung cancer that accounts for 30% of all lung cancer cases [3]. Numerous efforts have been made to improve the diagnosis and prognosis of LA. With timely treatment, about 56% of localized LA patients can survival more than 5 years [4]. However, only less than 16% of LA patients are detected at early stages due to the lack of sensitive marker, and distant tumor metastasis, which is closely correlated with death, is common by the time of diagnosis [5–8].

LA is frequently misdiagnosed as other clinical disorders, such as gastric reflux disease (GRD), chronic obstructive pulmonary disease (COPD) and asthma [9,10]. Misdiagnosis of LA leads to delayed treatment and high risk of death. Even with similar symptoms, different diseases have different molecular mechanisms with different molecular factors involved [11,12]. Therefore, certain differentially expressed molecular players may serve as potential diagnostic markers for LA. Long non-coding RNAs (lncRNAs) do not directly code proteins and are mainly involved in the regulation of protein-coding genes [13]. Certain lncRNAs have been reported to be associated with diagnostic and therapeutic potentials for cancers [14]. However, the roles of most lncRNAs in cancer are unclear. LncRNA FAM230B participates in gastric cancer and papillary thyroid cancer [15,16]. In both cancers, FAM230B can sponge miRNAs to upregulate the expression of downstream oncogenes to promote cancer progression [15,16]. However, its role in other cancers has not been reported. We performed preliminary sequencing analysis and observed the altered expression of FAM230B in LA, but not in other diseases that can mimic the symptoms of LA. We therefore speculated that FAM230B is involved in LA and its altered expression may serve as a potential biomarker to improve the diagnostic accuracy of LA. This study was then carried out to explore the role of FAM230B in the prognosis and diagnosis of LA.

## Materials and methods

### Patients and controls

Research subjects of the present study included 60 LA patients (40 males and 20 females, 57.7 ± 6.7 years old), 60 GRD patients (40 males and 20 females, 57.4 ± 6.8 years old), 60 COPD patients (40 males and 20 females, 57.9 ± 6.8 years old), 60 asthma patients (40 males and 20 females, 57.5 ± 6.4 years old) and 60 healthy controls (40 males and 20 females, 57.3 ± 7.0 years old). All these participants were enrolled at the Third Affiliated Hospital of Chongqing Medical University from March 2014 to March 2016. The Ethics Committee of this hospital approved this study (Approval No. 637CMU). Inclusion criteria: 1) newly diagnosed cases or controls passed systemic physiological examination; 2) patients with no initiated therapy. Exclusion criteria: 1) recurrent cases; 2) patients with blood relationship; 3) patients complicated with other clinical disorders. Different groups of participants showed similar age and gender distributions. Other clinical disorders were excluded from all patients. Healthy controls passed systemic physiological examinations. All participants provided written form informed consent. Clinical data of LA patients were listed in Table 1.

**Table 1.** Chi-squared test was analysis of the associations between patients’ clinical data and FAM230B in plasma

Characteristics	n	FAM230B		*Chi-squared*	*P*
		High	Low		
**Gender**					
**Female**	20	12	8	1.2	0.27
**Male**	40	18	22		
**Age(years)**					
**<55**	29	13	16	0.6	0.44
**≥55**	31	17	14		
**Smoking**					
**No**	42	20	22	0.32	0.57
**Yes**	18	10	8		
**Differentiated degree**					
**High/Middle**	38	20	18	0.29	0.59
**Low**	22	10	12		
**Clinical stage**					
**I–II**	24	10	14	1.11	0.29
**Tumor size**					
**<5 cm**	27	7	20	11.38	<0.001
**≥5 cm**	33	23	10	**≥5 cm**	33

### Sample collection

Tissue samples, including LA cancer and non-tumor tissues, were prepared by either dissecting the resected tumors from patients, or performing biopsies on patients who were not appropriate for surgical resection. All tissue samples were subjected to confirmation with histopathological analysis to make sure all samples were correct. On the day of admission, fasting blood was obtained from all participants. Blood samples were centrifuged at 1,200 g in a cold room to separate plasma samples, which were the supernatant after centrifugation. All samples were kept in liquid nitrogen prior to the following assays.

### RNA isolation and purification

NucleoSpin RNA Plus (Takara Bio) was applied to extract total RNAs from samples following the manufacturer’s instruction. gDNA Removal Column was used to remove genomic DNA in RNA samples. After RNA elution using nuclease-free water, RNA concentration and integrity were determined using 2100 Bioanalyzer. All RNA samples had satisfactory integrity (RIN value > 8.5).

### RT-qPCR

About 1 μg RNA sample was used as template to prepare cDNA samples through reverse transcriptions (RTs), which were performed using the Invitrogen SuperScript IV RT system. qPCR (20 ul system) was performed to detect the expression of FAM230B with 18S rRNA as an internal control. Ct values were collected. Three technical replicates were included. qPCRs were repeated if the difference of Ct values of three replicates was bigger than 0.5. The 2^−∆∆CT^ method was used to calculate the relative gene expression levels [17]. Primer sequences were: 5’-CTGAGAGACAGGGAACAGG-3’ (forward) and 5’-TCCTTGGAGCTTGACCTTG-3’ (reverse) for FAM230B; 5’-GTAACCCGTTGAACCCCATT-3’ (forward) and 5’-CCATCCAATCGGTAGTAGCG-3’ (reverse) for 18S rRNA. PCR cycling conditions were: 95°C for 45s, then 40 cycles of 95°C for 10s and 95°C for 40s.

### Statistical analysis

GraphPad Prism 6 software was used to compare datasets and plot images. Two groups were compared by Students’ t test. All datasets passed normal distribution test. The 60 LA patients were divided into high and low FAM230B level groups (n = 30, cutoff value = median expression level of FAM230B) Associations between patients’ clinical data and FAM230B in plasma were subjected to Chi-squared test. Diagnostic value of FAM230B for LA was analyzed with ROC curve analysis [18], in which LA patients were true positive cases, and the true negative cases were GRD, COPD, asthma, or control subjects. Using the 5-year follow-up data, survival curves were plotted for both high and low FAM230B groups. Log-rank test was used to compare survival curves. P < 0.05 was statistically significant.

## Results

### The expression of FAM230B in tissue and plasma samples

**Figure 1.**
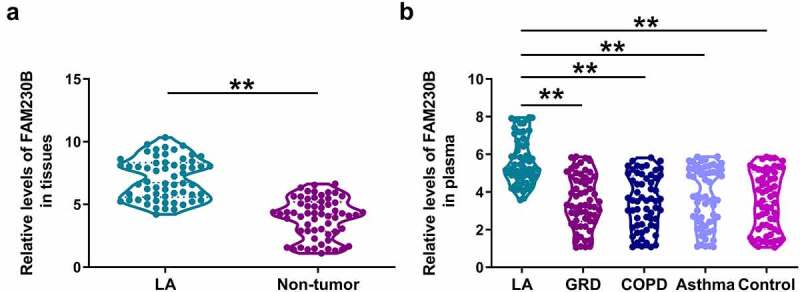
Analysis of FAM230B expression in tissue and plasma samples Tissue samples (paired LA and non-tumor tissues) from the 60 LA patients and plasma samples from GRD (n = 60), COPD (n = 60), asthma (n = 60), or control (n = 60) subjects were used to isolate total RNAs, which were subjected to RT-qPCR to determine the expression of FAM230B. FAM230B expression was compared between LA and non-tumor tissues (a), and among plasma samples from LA, GRD, COPD and asthma patients (b), **, *p* < 0.01.

Altered accumulation is an indicator of the involvement of a molecular factor in human diseases. Tissue samples (paired LA and non-tumor tissues) from the 60 LA patients and plasma samples from GRD (n = 60), COPD (n = 60), asthma (n = 60), or control (n = 60) were subjected to RNA isolation and RT-qPCR to determine the expression of FAM230B. The results showed that FAM230B was highly upregulated in LA tissues compared to that in non-tumor samples (Figure 1(a), *p* < 0.01). The expression levels of plasma FAM230B were specifically increased in LA patients, but not in GRD, COPD and asthma patients (Figure 1(b), *p* < 0.01). Therefore, FAM230B was specifically upregulated in LA, but not other lung diseases.

### Associations between patients’ clinical data and plasma FAM230B

To explore the possible function of FAM230B in LA, the 60 LA patients were divided into high and low FAM230B levels groups (n = 30, cutoff value = median expression level of FAM230B). Associations between patients’ clinical data and FAM230B in plasma were subjected to Chi-squared test analysis. It showed that plasma FAM230B was closely correlated with tumor size, but not other clinical factors of LA patients (Table 1). Therefore, FAM230B was likely involved in tumor growth, but not other aspects of LA.

### Correlations between plasma FAM230B and FAM230B in tissue samples

**Figure 2.**
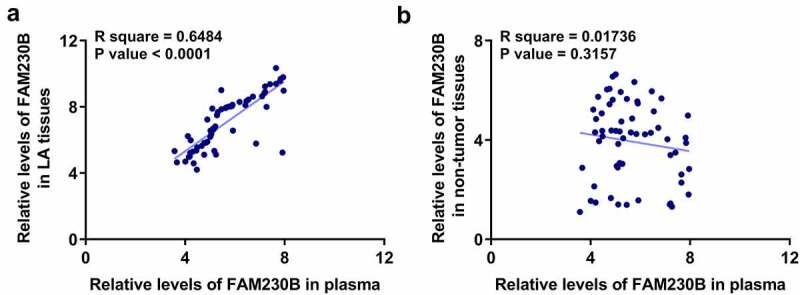
Correlations between plasma FAM230B and FAM230B in tissue samples Correlations between plasma FAM230B and FAM230B in LA (a) or non-tumor (b) tissue samples were analyzed with Pearson’s correlation coefficient.

To explore where plasma circulating FAM230B from, the correlations between plasma expression levels of FAM230B and the expression of FAM230B in LA or non-tumor tissue samples were analyzed with Pearson’s correlation coefficient. It was observed that plasma expression of FAM230B was closely and positively correlated with FAM230B in LA tissue samples (Figure 2(a)). However, plasma expression of FAM230B was not closely correlated with FAM230B in non-tumor tissues (Figure 2(b)). Therefore, plasma FAM230B was likely from LA tissues.

### Diagnostic value of plasma FAM230B for LA

**Figure 3.**
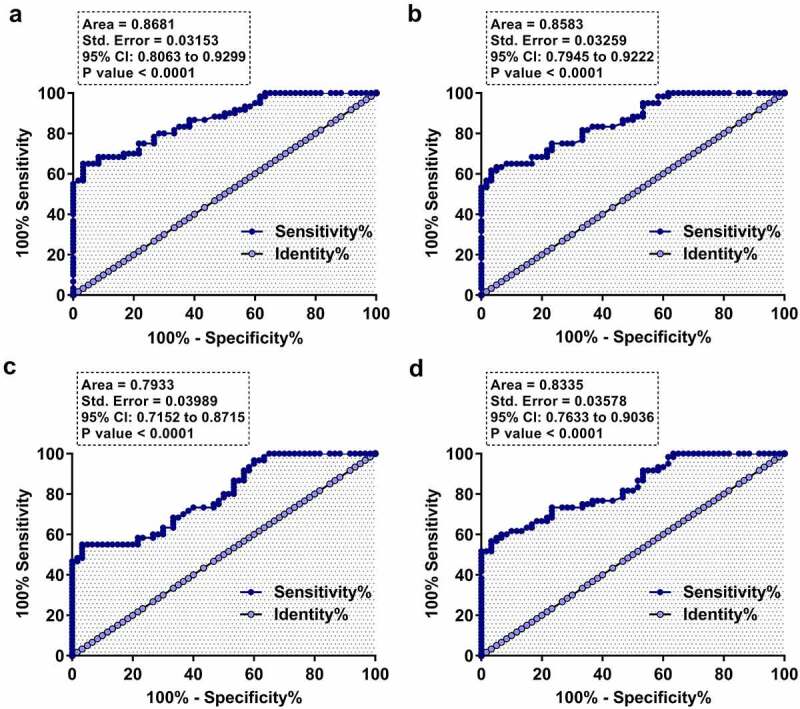
Diagnostic value of plasma FAM230B for LA Diagnostic value of FAM230B for LA was analyzed with ROC curve analysis, in which LA patients were true positive cases, and the true negative cases were GRD (a), COPD (b), asthma (c), or control (d) subjects.

Diagnostic value of FAM230B for LA was evaluated with ROC curve analysis, in which LA patients were true positive cases, and the true negative cases were GRD, COPD, asthma, or control subjects. It was observed that increased plasma expression levels of FAM230B effectively separated LA patients from GRD (Figure 3(a)), COPD (Figure 3(b)), asthma (Figure 3(c)), or the control (Figure 3(d)) subjects. Therefore, plasma FAM230B may be applied in the diagnosis of LA.

### Prognostic value of plasma FAM230B for LA

**Figure 4.**
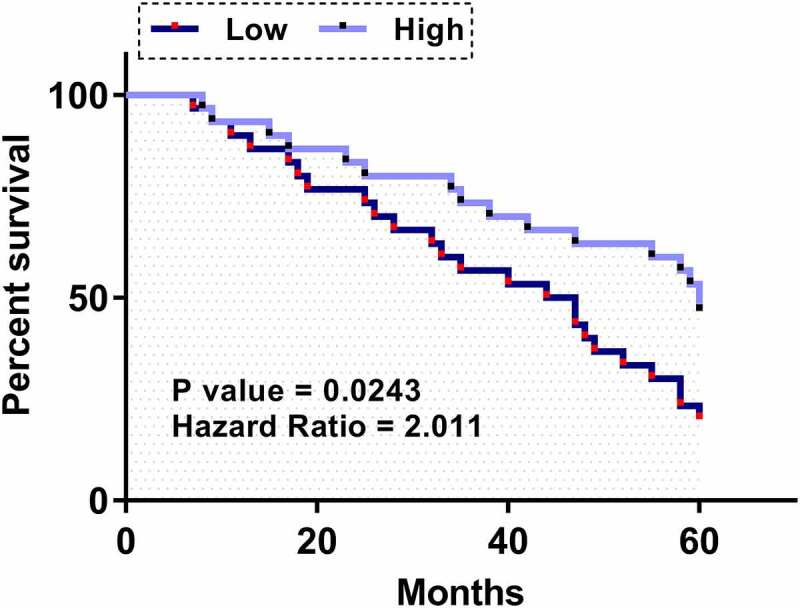
Prognostic value of plasma FAM230B for LA Survival analysis was performed by carrying out a 5-year follow-up study, followed by a survival curve analysis. The 60 LA patients were divided into high and low FAM230B levels groups (n = 30, cutoff value = median plasma expression level of FAM230B). Using the 5-year follow-up data, survival curves were plotted for both high and low FAM230B groups. Log-rank test was applied to compare survival curves.

To explore the prognostic value of plasma FAM230B for LA, the 60 LA patients were divided into high and low FAM230B level groups (n = 30, cutoff value = median plasma expression level of FAM230B). Using the 5-year follow-up data, survival curves were plotted for both high and low FAM230B level groups. Log-rank test was applied to compare survival curves. High expression levels of plasma FAM230B were closely correlated with poor survival (Figure 4). Therefore, plasma expression levels of FAM230B might be applied in the diagnosis of LA.

## Discussion

In this study the involvement of lncRNA FAM230B in LA was explored. We analyzed the expression pattern of FAM230B in LA and our results demonstrated that this lncRNA can be applied to improve the diagnosis of LA.

FAM230B is a newly identified lncRNA with known functions only in gastric cancer and papillary thyroid cancer [15,16]. FAM230B was highly upregulated in gastric cancer and it upregulates TOP2A by sponging miR-27a-5p to promote both tumor growth and metastasis [15]. FAM230B is also highly expressed in papillary thyroid cancer and interacts with the miR-378a-3p/WNT5A axis to accelerate tumor metastasis [16]. Our study observed the upregulation of FAM230B in LA tissues compared to that in non-tumor tissues, suggesting the oncogenic role of FAM230B in LA. Although we did not explore the role of FAM230B in the progression of LA, our association analysis showed that plasma expression of FAM230B was only closely correlated with tumor size, but not other factors. Therefore, FAM230B is likely involved in tumor growth of LA. However, this speculation should be verified by *in vitro* cell and/or *in vivo* animal model experiments.

Interestingly, plasma FAM230B is only upregulated in LA, but not in GRD, COPD and asthma patients, compared to that in the controls. The expression of FAM230B in plasma is only closely correlated with FAM230B in LA tissues, but not FAM230B in non-tumor tissues, suggesting that plasma FAM230B in LA patients is mainly from LA tissues. High expression levels of FAM230B in LA plasma effectively separated LA patients from the controls. Therefore, measuring the expression levels of plasma FAM230B prior to treatment may increase the accuracy of LA diagnosis and reduce the rate of misdiagnosis. However, we only included 60 patients in each group. Therefore, future studies may carry a similar ROC curve analysis with a much bigger sample size to further confirm our conclusions. Our study also showed the close correlated between high expression levels of plasma FAM230B and the worse overall survival of LA patients. Measuring the plasma expression levels of FAM230B may help the identification of patients with high risk of deaths in short-term, thereby apply interventions to prolong survival.

Although previous studies have characterized a considerable number of biomarkers for LA [19,20], most of these studies only enrolled healthy controls as true negative cases in ROC curve analysis. However, LA in clinical practice can be misdiagnosed as other diseases that can mimic the symptoms of LA. Our study is the first to characterize a biomarker that can be applied to separate LA patients from GRD, COPD, asthma and the control groups. The main limitation of this study is the small sample size. Therefore, the role of FAM230B in the diagnosis and prognosis of LA should be further explored by studies with a bigger sample size. Moreover, the function of FAM230B in LA and the mechanisms that mediate the function remain to be explored.

## Conclusion

In conclusion, FAM230B is upregulated in LA and it may serve as a potential diagnostic and prognostic biomarker for LA. Our study is the first to characterize a biomarker that can be applied to separate LA patients from patients with GRD, COPD or asthma. However, further validations are still needed.

## Data Availability

The data that support the findings of this study are available on request from the corresponding author.

## Abbreviations

Lung adenocarcinoma (LA)

Gastric reflux disease (GRD)

Chronic obstructive pulmonary disease (COPD)
